# Using New Camera-Based Technologies for Gait Analysis in Older Adults in Comparison to the Established GAITRite System

**DOI:** 10.3390/s20010125

**Published:** 2019-12-24

**Authors:** Anika Steinert, Igor Sattler, Karen Otte, Hanna Röhling, Sebastian Mansow-Model, Ursula Müller-Werdan

**Affiliations:** 1 Geriatrics Research Group, Charité–Universitätsmedizin Berlin, corporate member of Freie Universität Berlin, Humboldt-Universität zu Berlin, and Berlin Institute of Health, Charitéplatz 1, 10117 Berlin, Germany; igor.sattler@charite.de (I.S.); ursula.mueller-werdan@charite.de (U.M.-W.); 2 NeuroCure Clinical Research Center, Charité-Universitaetsmedizin Berlin, corporate member of Freie Universität Berlin, Humboldt-Universität zu Berlin, and Berlin Institute of Health, Charitéplatz 1, 10117 Berlin, Germany; karen.otte@motognosis.com; 3 Motognosis GmbH, Schönhauser Allee 177, 10119 Berlin, Germany; hanna.roehling@motognosis.com (H.R.); smm@motognosis.com (S.M.-M.)

**Keywords:** gait analysis, movement, older adults, gait parameters, mobile technologies

## Abstract

Various gait parameters can be used to assess the risk of falling in older adults. However, the state-of-the-art systems used to quantify gait parameters often come with high costs as well as training and space requirements. Gait analysis systems, which use mobile and commercially available cameras, can be an easily available, marker-free alternative. In a study with 44 participants (age ≥ 65 years), gait patterns were analyzed with three different systems: a pressure sensitive walkway system (GAITRite-System, GS) as gold standard, Motognosis Labs Software using a Microsoft Kinect Sensor (MKS), and a smartphone camera-based application (SCA). Intertrial repeatability showed moderate to excellent results for MKS (ICC(1,1) 0.574 to 0.962) for almost all measured gait parameters and moderate reliability in SCA measures for gait speed (ICC(1,1) 0.526 to 0.535). All gait parameters of MKS showed a high level of agreement with GS (ICC(2,k) 0.811 to 0.981). Gait parameters extracted with SCA showed poor reliability. The tested gait analysis systems based on different camera systems are currently only partially able to capture valid gait parameters. If the underlying algorithms are adapted and camera technology is advancing, it is conceivable that these comparatively simple methods could be used for gait analysis.

## 1. Introduction

Falls are amongst the most serious and costly accidents in older adults. Due to problems in defining and recording falls, prevalence and incidence data vary widely. According to data from the World Health Organization, 28% to 35% of adults aged over 65 years fall each year. From the age of 70, this proportion rises up to 42% [1]. A survey among participants aged 65 to 89 years showed an incidence of 38.7 female and 29.7 male fallers per 100 persons per year [2]. Especially in old age, falls may have far-reaching consequences, such as fractures and hospital admissions. However, even falls without injury often have serious consequences. The fear of further falls is a major cause of actual future falls and it decreases confidence in one’s own mobility, which can lead to a massive reduction in quality of life [3].

A comprehensive gait analysis can reveal deviations and impairments in gait behavior and enable medical health professionals to assess the risk of illness or injury. The measured parameters provide useful diagnostic and therapeutic information when measured accurately. According to a review by Springer and Seligmann, the most relevant parameters in gait analysis are gait speed, step length, step time, cadence (number of steps per minute), and stance time [4]. Age-related changes in step parameters are associated with a decrease in gait symmetry and step length and an increase in step time and step width [5]. The risk of falling increases with a reduction in gait speed. Even a gait speed reduction of only 0.1 m/s is related to a 7% increase in the risk of falling. If the gait speed is below 0.7 m/s, the risk of falling is 1.5 times higher compared to participants with a normal gait speed (>1.0 m/s) [6].

One of the most recognized gold standards for gait analysis is the GAITRite system (GS), a portable gait analysis tool for the automated measurement of gait parameters. Studies could classify the use of GS as valid and reliable [7]. However, its use is limited due to the need for expensive devices that are not always available in the clinical environment. Furthermore, only step parameters can be captured, and thus full-body gait analysis is not possible. Recent findings indicate that Microsoft Kinect offers a cost-effective method for gait analysis [8,9]. However, studies have shown that Kinect is only a valid measuring instrument for some gait parameters [4].

In a validation study using the GAITRite system as a reference system, it was found that Kinect is well suited to determine general parameters such as average stride length or average stride time (a Spearman correlation coefficient of 0.94 for average stride length and 0.75 for average stride time per stride). Determining more accurate results for individual strides is more difficult (SCC of 0.74 for stride length and 0.43 for stride time per stride), which increases the difficulty of determining more complex gait parameters such as gait symmetry [10]. In a study, Clark et al. also demonstrated limited validity of the Kinect system for complex gait parameters [11].

Because some of the established systems are expensive, need a large amount of space, lack validity, or need extensive training, new devices using relatively recent and widespread information and communication systems such as smartphones were developed. Within the presented study, two camera-based systems for measuring different gait parameters were validated against a gold standard for gait analysis.

## 2. Materials and Methods

### 2.1. Apparatus

The gait analysis was conducted with three different systems: a pressure sensitive walkway system (GAITRite-System, GS), Motognosis Labs Software using a Microsoft Kinect Sensor for Xbox One (Microsoft Kinect system, MKS), and a smartphone camera-based application (smartphone camera application, SCA).

(1) GS: GAITRite is a 5.2 m long (active length 4.27 m) and 90 cm wide (active wide 61 cm) carpet with 16,128 embedded sensors in a grid. The sensors are placed at a distance of 1.27 cm and are activated by mechanical pressure. GS allows the measurement of different temporal (e.g., step time, velocity, single/double support) and spatial (e.g., step length, stride length, distance) parameters. The carpet is connected to a computer via an interface cable. Prior to the gait analysis, the participant’s age, weight, height, and leg length (right and left) had to be entered manually. The validity of GS was previously investigated in several studies [7,12,13] and used as gold standard in the presented study.
(2) MKS: Motognosis Labs is software developed for the motor assessment of patients with neurodegenerative diseases [14] using a consumer 3D camera (Microsoft Kinect V2) to collect depth silhouettes of individuals (visual perceptive computing). The Software Development Kit of Microsoft (SDK V14.09) uses artificial intelligence to locate 25 different anatomical landmarks [15], which are then used by the software to calculate movement kinematics similar to GS. The system was placed at the end of GS with 1.7 m distance to the edge of the carpet. The measurement range of the system is up to 4.5 m, limiting the area covered by GS and MKS to approximately 3 m.
(3) SCA: The smartphone application conducts gait analysis by recording a video of the subject with a 2D smartphone camera and an underlying algorithm. Within the smartphone application participants’ age, sex, height, and weight must be entered. SCA applies the recent advances of artificial intelligence to the problem of human pose estimation. Using a 2D smartphone camera and a deep convolutional neural net, the application estimates a 3D skeletal model based on a video of a person walking. The underlying algorithm was developed using the VNect algorithm (3D joint and skeleton detection). The VNect algorithm is a real-time method, which captures the full global 3D skeletal pose of human using a single RGB camera [16]. SCA was installed on a Nexus 5 smartphone (Android).

### 2.2. Procedure

The inclusion criteria for participants were a minimum age of 65 years and participants’ signed informed consent. Exclusion criteria were severe cognitive, sensory, or motoric disorders, and a legal representative. Participants were recruited from former contacts of the Geriatrics Research Group, Charité–Universitätsmedizin Berlin. In an initial telephone screening, participants were informed about the study procedure, the inclusion and exclusion criteria were checked, and an appointment for the gait analysis was arranged (minimum of 24 h after screening).

**Figure 1:**
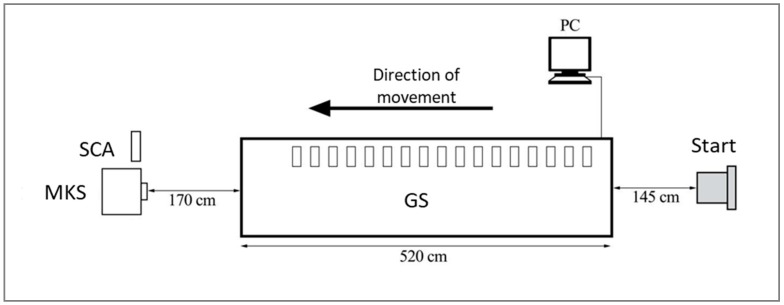
Experimental setup.

The gait analysis was conducted in the laboratory of the Geriatrics Research Group in Berlin. Firstly, participants were once again informed about the study procedure and data protection. After giving their informed consent, participants had to complete a questionnaire with regard to socio-demographic data (age, sex, weight, height, leg length; self-deployed questionnaire). Subsequently, each participant was asked to complete six walks with two speed conditions: preferred gait speed (PGS) and fast gait speed (FGS). This resulted in 264 walks overall (44 participants × 6 walks). For the first three walks, participants were instructed to walk over the carpet (GS) beyond the end at a self-selected normal speed. The following three walks should be completed at an FGS (instruction: a velocity that is faster than you would walk normally). For each walk, GS was first started at the computer and then MKS and SCA were initiated, before the participant’s walk, so that all three systems analyzed the subject’s gait simultaneously (Figure 1). GS measurement was initiated by the first pressure contact to the carpet. The video recording of MKS and SCA began when the participants entered the recorded area. After each walk, study personnel verified that the systems were measuring correctly. At the end, the participants received a short evaluation of their gait pattern on the basis of the GAITRite data. The study visit took approximately one hour for the participants. Both the ethics committee and the data protection office of the Charité approved all study procedures.

### 2.3. Data Analysis

The data of the three systems were synchronized in an excel file. Four gait parameters were compared between the three systems: gait speed, cadence, step length (right/left), and step time (right/left). The four relevant gait parameters were calculated from all three systems for all participants and manually merged by the study personnel into an Excel file. The parameters were output in different units of measurement (e.g., gait speed in km/h or m/s). Accordingly, the data is partially converted. For quality assurance purposes, 10% of the data records were randomly checked by an external scientific employee. All gait parameters were tested for normality using a Kolmogorov-Smirnov (K-S) test under both conditions (PGS, FGS). The mean values of three walks per condition were used to compare the systems. To identify the retest reliability for measurement repetitions for each system, intraclass correlation coefficients (ICC(1,1); one-way random effects) were calculated. Using mean values of three walks, ICCs(2,k) (two-way random effects, absolute agreement, single rater/measurement) with 95% confidence intervals and Pearson Correlation were calculated to identify the level of agreement between two systems. According to Koo et al., values less than 0.5, between 0.5 and 0.75, between 0.75 and 0.90, and values higher than 0.90 indicate poor, moderate, good, or excellent reliability, respectively [17].

## 3. Results

### 3.1. Subjects

Forty-four older adults participated in the study. Participants were on average 73.9 years old (±6.0 years, range: 65 to 91 years). Half of the participants (n = 22) were female. For gait analysis, height, weight, and leg length were measured (Table 1). The mean height and weight were 168.8 cm (±8.9 cm) and 76.2 kg (±15.5 kg), respectively.

### 3.2. Concurrent Validity

Table 2 reports the mean values and differences between the systems for all measured gait parameters for GS, MKS, and SCA under both conditions, the standard deviation for mean values, and the 95% confidence intervals of average differences between both systems. Recorded mean values differed in both conditions between GS and MKS for gait speed and step length (right). The largest differences were recorded for gait speed in FGS (Diff = 5.76 cm/s) and PGS (Diff = 4.01 cm/s). Furthermore, there were significant differences between GS and MKS considering step time (left) and step time (right) in PGS. However, GS and MKS showed a strong positive correlation for all gait parameters and both conditions (0.845 ≤ r ≤ 0.988).

The ICC(2,k) showed a high level of agreement between GS and MKS for all gait parameters in PGS and FGS, ranging from 0.811 to 0.981. Comparing mean values for GS and SCA, significant differences were observed in almost all gait parameters, except gait speed in PGS and step length (left and right) in FGS. Larger differences were observed in the comparison of GS and SCA. In particular, the cadence was substantially higher when measured with the SCA in PGS (Diff = −42.23 steps/min) and FGS (Diff = 46.08 steps/min). Only small to medium correlations existed between the mean values of GS and SCA. With values less than 0.5, the ICC(2,k) indicates poor agreement between GS and SCA for all gait parameters and both conditions (Table 2).

### 3.3. Intertrial Repeatability

The repeatability of the recorded gait parameters of the three systems under both conditions was analyzed using ICC(1,1) (Table 3). All gait parameters measured by GS were found to have good reliability in PGS (ICC(1,1): 0.786 to 0.860) and moderate reliability in FGS (ICC(1,1): 0.502 to 0.721) except in step time (right) and cadence. All gait parameters measured by MKS showed good to excellent reliability in FGS (ICC(1,1): 0.809 to 0.962). Gait parameters in PGS showed good to moderate reliability, except step time (right and left; ICC (1;1): 0.426 and 0.453). SCA was found to have moderate reliability in gait speed under both conditions (ICC(1,1): 0.526 to 0.535). Other parameters measured by the SCA showed poor reliability (ICC(1,1): 0.125 to 0.368).

## 4. Discussion

This study investigated the validity of camera-based technologies for gait analysis in comparison to a well-established gait analysis system. Accordingly, 264 walks of 44 older adults at two walking speed conditions (PGS and FGS) were recorded with three systems. As already shown in other studies, GS measures of gait parameters showed good reliability for PGS, which was confirmed for gait parameters in the present study, but only moderate in FGS [7,13]. To a large extent, good reliability was also demonstrated for MKS in the present study. Between the included MKS and GS, a good level of agreement was found, especially in PGS. In contrast, a comprehensive review by Springer and Seligman concluded that gait analysis systems based on a Microsoft Kinect for spatiotemporal gait parameters had limited validity [4].

The mean values of the SCA already differ to a high extent from the mean values of GS and MKS. This was also found in the ICC results, in which only a poor level of agreement for all gait parameters at both speed conditions was measured. Furthermore, the intertrial repeatability was only moderate for one of the six included gait parameters.

The usage of a smartphone camera for the gait analysis of spatiotemporal parameters has not yet been extensively investigated. The study of Finkbiner et al. demonstrated significant moderate agreement in kinematic parameters (knee angles) measured by an application using a smartphone camera and a 3D motion capturing system [18]. Furthermore, some recent studies have investigated the use of smartphone motion sensors (e.g., accelerometer) to capture and analyze movements [19,20]. In this context, Manor et al. found significant correlations in stride length between the GAITRite system and a self-developed iPhone application [19].

To conclude, even if smartphones are comparatively cheap, mobile, and easily available, the smartphone camera-based application used in the present study was not able to detect relevant gait parameters in the same manner as an already well-established system. The main reason for this might be the underlying algorithm, which analyzes the sensor data and the used 2D camera. This algorithm must be improved, and studies demonstrating the validity and reliability of the system must be conducted before using SCA for gait analysis in clinical situations. In contrast, the collected data of the MKS showed already high agreement with the data of the gold standard in the majority of the measured gait parameters. In further studies, it is important to also include participants with physical impairments and/or walking aids as the target group for which gait analysis systems are usually made.

## 5. Perspectives

GS data will be used to help developers to adapt to the requirements for systematic gait analysis, which is particularly used in medicine and physiotherapy. Based on the presented study, the algorithms of MKS and SCA will be further developed. In particular, the present study is used as a first basis for strengthening the necessary data science experience in the further development of SCA analysis. Data scientists will continue to develop underlying AI based calculation methods to improve the accuracy of SCA gait analysis. Compared to gait analysis with a Microsoft Kinect camera, which was validated with different target groups in previous studies, gait analysis with a smartphone camera is a completely new method, whose underlying artificial intelligence must first be learned before further studies can be carried out.

## Figures and Tables

**Table 1:** Socio-demographic data of participants.

	Total	Female	Male
N	44	22	22
Age [Mean ± SD, years]	73.9 ± 6.1	74.1 ± 6.1	73.7 ± 6.2
Height [Mean ± SD, cm]	168.8 ± 8.9	162.4 ± 6.2	175.1 ± 6.2
Weight [Mean ± SD, kg]	76.2 ± 15.5	68.3 ± 14.0	84.1 ± 12.8
Leg length right [Mean ± SD, cm]	90.1 ± 5.4	87.4 ± 4.9	92.8 ± 4.6
Leg length left [Mean ± SD, cm]	89.9 ± 5.5	87.1 ± 5.2	92.7 ± 4.5

Note: SD = standard deviation.

**Table 2:** Mean difference, SD of the differences, 95% confidence interval, and t-test values for GS and MKS and GS and SCA at preferred and fast speed.

		GS	MKS	SCA	GS-MKS				GS-SCA			
		Mean (SD)	Mean (SD)	Mean (SD)	Diff [95% CI]	*t*-Test *p*-Value	Pearson Corr.	ICC(2,k)	Diff [95% CI]	*t*-Test *p*-Value	Pearson Corr.	ICC(2,k)
Preferred speed	Gait speed [cm/s]	120.69 (19.90)	116.69 (19.04)	122.05 (24.81)	4.01 [3.14; 4.88]	0.000	0.988	0.981	−0.44 [−7.89; 7.02]	0.906	0.275	0.434
	Cadence [steps/min]	111.90 (8.72)	111.75 (12.23)	155.51 (23.86)	−1.16 [−2.81; 0.49]	0.162	0.876	0.925	−42.23 [−48.25; −36.21]	0.000	0.078	0.020
	Step length, left [cm]	64.73 (7.78)	62.45 (8.00)	51.94 (25.04)	1.99 [1.16; 2.82]	0.000	0.952	0.958	14.79 [10.15; 19.43]	0.000	0.316	0.233
	Step length, right [cm]	65.04 (7.92)	62.38 (8.94)	60.10 (41.84)	2.59 [1.67; 3.51]	0.000	0.946	0.941	8.35 [2.77; 13.93]	0.004	0.413	0.394
	Step time, left [s]	0.54 (0.05)	0.55 (0.08)	0.62 (0.24)	−0.01 [−0.02; 0.01]	0.307	0.887	0.921	−0.08 [−0.13; −0.03]	0.004	0.295	0.222
	Step time, right [s]	0.54 (0.04)	0.53 (0.06)	0.67 (0.39)	0.01 [0.01; 0.02]	0.003	0.857	0.903	−0.10 [−0.17; −0.03]	0.004	0.158	0.090
Fast speed	Gait speed [cm/s]	164.80 (23.40)	158.70 (22.20)	143.60 (37.00)	5.76 [2.59; 8.93]	0.001	0.904	0.922	19.31 [9.86; 28.77]	0.000	0.424	0.494
	Cadence [steps/min]	132.70 (10.20)	129.60 (14.80)	177.50 (28.60)	−2.16 [−4.77; 0.45]	0.097	0.876	0.904	−46.09 [−52.43; −39.74]	0.000	0.409	0.119
	Step length, left [cm]	74.50 (9.30)	72.30 (10.40)	79.60 (52.50)	1.42 [−0.28; 3.12]	0.095	0.910	0.947	−2.72 [−12.10; 6.65]	0.560	0.466	0.389
	Step length, right [cm]	74.10 (8.60)	70.00 (10.20)	68.20 (30.30)	1.43 [0.30; 2.56]	0.016	0.958	0.971	2.31 [−3.73; 8.35]	0.442	0.342	0.415
	Step time, left [s]	0.45 (0.03)	0.47 (0.05)	0.54 (0.22)	0.01 [0.00; 0.03]	0.101	0.845	0.811	−0.06 [−0.10; −0.01]	0.013	0.267	0.176
	Step time, right [s]	0.45 (0.03)	0.46 (0.06)	0.61 (0.45)	0.00 [−0.02; 0.02]	0.873	0.884	0.864	−0.09 [−0.15; −0.03]	0.006	−0.137	−0.077

Note. SD = standard deviation, Diff [95% CI] = Differences in mean values with confidence intervals lower and upper bound, ICC = intraclass correlation coefficient.

**Table 3:** ICC(1,1) Intratrial repeatability for GS, MKS, and SCA at preferred and fast speed.

		GS		MKS		SCA	
		SEM	ICC(1,1)	SEM	ICC(1,1)	SEM	ICC(1,1)
Preferred speed	Gait speed [cm/s]	1.739	0.816	1.664	0.823	2.219	0.535
	Cadence [steps/min]	0.768	0.834	1.136	0.574	2.134	0.298
	Step length, left [cm]	0.685	0.854	0.749	0.843	2.258	0.125
	Step length, right [cm]	0.697	0.860	0.849	0.646	3.758	0.225
	Step time, left [s]	0.004	0.826	0.008	0.453	0.022	0.142
	Step time, right [s]	0.004	0.786	0.006	0.426	0.036	0.100
Fast speed	Gait speed [cm/s]	2.076	0.502	3.285	0.944	1.980	0.526
	Cadence [steps/min]	0.909	0.349	2.539	0.901	1.663	0.368
	Step length, left [cm]	0.814	0.508	4.692	0.962	1.108	0.136
	Step length, right [cm]	0.757	0.721	2.750	0.893	1.103	0.177
	Step time, left [s]	0.003	0.508	0.020	0.835	0.007	0.160
	Step time, right [s]	0.003	0.488	0.042	0.809	0.008	0.148

Note. ICC = intraclass correlation coefficient, GS = GAITRite system, MKS = Microsoft Kinect system, SCA = smartphone camera application, SEM = standard error of measurement.
